# Using Sequential Email Messages to Promote Health Behaviors: Evidence of Feasibility and Reach in a Worksite Sample

**DOI:** 10.2196/jmir.8.1.e3

**Published:** 2006-03-30

**Authors:** Patricia D Franklin, Paula F Rosenbaum, Michael P Carey, Michael F Roizen

**Affiliations:** ^4^Cleveland Clinic FoundationDivision of AnesthesiaCritical Care Medicine and Comprehensive Pain ManagementClevelandOHUSA; ^3^Center for Health and BehaviorSyracuse UniversitySyracuseNYUSA; ^2^Center for Outcomes Research and EvaluationSUNY Upstate Medical UniversitySyracuseNYUSA; ^1^Department of OrthopedicsUniversity of Massachusetts Medical SchoolWorcesterMAUSA

**Keywords:** Health promotion, employee wellness, information technology in health care

## Abstract

**Background:**

US adults report suboptimal physical activity and fruit and vegetable intake. Innovative strategies to promote healthy behaviors are needed. Employee health promotion programs have been associated with reductions in health risks but are labor-intensive and costly to implement. Email and Web-based worksite programs have the potential to reach a broad adult population and to provide a cost-effective approach to employee wellness programming.

**Objective:**

To assess the feasibility of using sequential email messages to promote physical activity and increase fruit and vegetable intake among employed adults.

**Methods:**

Employees at one worksite of a large insurance company in New York State were invited to participate. Interested workers provided written consent. After completing a baseline survey, participants received daily emails, Monday through Friday, for 26 weeks. The emails provided (a) succinct strategies to encourage physical activity or increase fruit and vegetable intake and (b) links to detailed Web-based information and tools. Program reach was assessed by the number of emails opened, measures of sustained participation over 6 months, and the number of health-related Web-links clicked.

**Results:**

Of 960 employees, 388 (40%) consented to participate; of these, 345 (89%) completed the baseline health survey. After 6 months, 70% of the 345 participants had opened 50% or more of the daily emails. In addition, 75% of participants continued to open at least one email a week through week 26 of the study. Email opening rates did not vary by gender, age, income, education, ethnicity, or baseline health behavior.

**Conclusions:**

The rate of enrollment and sustained participation document the feasibility, broad reach, employee acceptance, and potential value of using electronic communications for health promotion in the workplace.

## Introduction

### Does Email Have a Role in Worksite Health Promotion?

Employee health promotion programs have been associated with reductions in health risks. Pelletier [1] and Aldana [2] reviewed 11 and 72 studies, respectively, and found consistent evidence that worksite health promotion programs were associated with reductions in health risks and costs. However, traditional worksite health programs are labor intensive and costly to implement. In contrast, email and Web-based programs have the potential to reach a broad employee population with minimal delivery costs after the initial message development.

By design, the Internet spans geographic and time differences, sustains relationships based on interests, and provides links between people and information. In addition, email and Web access is available 24 hours/day, 7 days/week, and information can be customized to serve the individual characteristics of the user [3]. These attributes may serve both the employer and the employee as program access is available across work shifts and into vacation and leisure time. In 2005, a total of 74% of US adults reported having Web access, including 66% with home access and 36% with work access [4]. As well, 41% of employers report that they are “likely” to use Web-based education as a component of a health care utilization management program, and an additional 47% reported they were “somewhat likely” [5]. Thus, worksite leadership is poised to adopt email and Web-based programming, so it is timely to evaluate the reach and effectiveness for use in employee health promotion.

### Suboptimal Health Behaviors

Levels of physical activity and fruit and vegetable consumption are well below recommended guidelines and have remained substandard despite the health promotion efforts spurred by the Healthy People 2000 and 2010 initiatives. Specifically, 25% of Americans report no regular physical activity [6]. Between 1990 and 2004, the number of Americans who reported moderate levels of physical activity actually decreased from 23% to 15% in 2004. In a recent national survey, 85% of respondents reported fewer than 60 min/week of leisure time physical activity [7].

In addition to lack of physical activity, suboptimal diets contribute to the prevalence of overweight and obese adults: 77% of adults report diets that include less than the recommended daily intake of fruits, vegetables, and vitamins [8], and 55% report weights that categorize them as overweight, and the prevalence is increasing. Of all adults, 31% meet the definition of obesity, and this number is higher for women [9]. Recent analyses found employers spend an additional $462 to $2485 each year on medical expenditures and work absence for employees who are more than 30 pounds overweight [10]. Despite the costs associated with behavioral choices, less than 3% of health dollars are spent on public health efforts to improve health behaviors [11].

## Methods

This preliminary study evaluated participation and attrition rates over a 6-month, email-based health promotion program and the characteristics of employees who sustained participation. This study was the first phase in a worksite intervention trial designed to assess health behavior change following differing eHealth delivery modes of health-promoting materials. The Institutional Review Boards from collaborating institutions approved all procedures.

### Setting

This preliminary study was conducted at the main office of a large health insurance company in upstate New York. In August 2003, the worksite employed 960 full-time workers; an estimated 90% had computer access at their desks. For this study, employees without desktop computers were offered daily access to a central computer in the employee lunchroom or could identify a personal email address at which to receive emails. The total employee population was 76% female, 90% white, with a mean age of 43 years. Distribution of income was as follows: 24% earned less than $29999, 46% earned between $30000 and $49999, and 30% earned more than $50000. The organization employed building maintenance, clerical, customer service, actuarial, sales, information technology, and health professionals.

### Enrollment

The invitation to participate was initiated by an email from the company president to all employees, followed by announcements posted in employee elevators and a 5-minute presentation at the quarterly “all-employee” meeting. A series of nine midday onsite study enrollment sessions were scheduled across 3 weeks. Enrollment was held in the employee lunchroom for convenience. The employee health office distributed reminder emails on each enrollment day in order to encourage participation. After a consent form was signed, a unique research ID number was assigned to each employee for use with all study related documents. Participants provided their preferred email address and work telephone number on the consent form for use by the study team. Both full- and part-time employees were included because it was not necessary for the email to be read on the same day it was delivered.

### Health Assessment

In order to evaluate employee characteristics associated with participation and to validate the survey to be used in the subsequent trial, all consenting employees in the preliminary study completed a baseline health assessment prior to the start of the email health promotion program. This included validated assessments of demographic variables, exercise (International Physical Activity Questionnaire [12]), fruit and vegetable consumption (Quick Food Scan from the National Cancer Institute [13]), antecedents of health behavior change [14], intention to change, health status (e.g., Short Form 12) [15], and health care utilization. The employer agreed to allow participants to complete the 30-minute assessment during work hours. Reminder emails prompted participants to complete the assessment within 7 days and to return it to confidential study bins in the worksite mailroom. The employee health office collected the forms daily for secure storage, and the study staff collected assessments each week.

### Email Messages

Following completion of the baseline health assessment, employees received an email with an explanation of the health promotion program. The email address provided on the enrollment form was used for this contact. If the email was returned as “undeliverable,” a study coordinator telephoned the employee to verify participation and the email address. Approximately 5% of email addresses needed correction.

**Figure 1:**
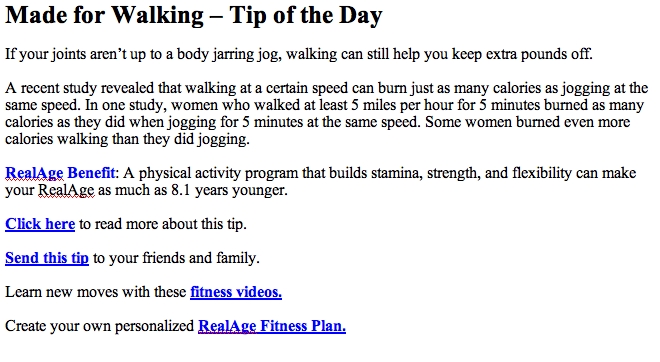
Sample email health message

Daily health tips (129 total) were delivered from an established website (RealAge.com) Monday through Friday for 26 weeks, starting in October 2003. No marketing messages were included in study emails. Approximately 30% of emails addressed fruit and vegetable intake, 47% addressed muscle strength and aerobic activity, and 23% addressed general healthy living. Tips were grouped by these topics and rotated throughout the 26-week period (i.e., week 1 addressed fruit and vegetable intake, week 2 addressed aerobic activity, and so forth). Each email emphasized the gains associated with healthy habits and included three components: (1) a specific diet or exercise tip, (2) an estimate of the number of “RealAge” years younger associated with adopting the behavior [16], and (3) embedded links for self-monitoring tools and additional information (Figure 1). For example, the diet and nutrition emails included serving tips, recommended seasonal fruits, and Web links to recipes, personal calorie counters, and further nutrient information. Physical activity emails suggested alternative ways to incorporate exercise into daily routines, while Web links offered exercise planning and tracking tools.

### Measures of Participation

Process measures of program participation included (a) total number of emails opened, (b) sustained participation over 6 months (i.e., number of days on study and the frequency of opening ≥ 4 emails per week), and (c) the use of health-related Web links. To assess heterogeneity, measures of participation were evaluated by employee demographic characteristics such as gender, age, and education.

To facilitate the evaluation of employee participation by the study team, the RealAge server tracked the use of email messages and Web links. When a participant “clicked” to open an email or link, the RealAge server delivered an HTML version of the message. Thus, the server recorded only HTML-delivered messages as “open” messages. These were recorded by study participant and by date of message. If a participant previewed the email but did not “click” to open it, the email was not counted as open.

To calculate an open rate, the number of HTML-version messages was divided by the total number of emails sent. Open rates were associated with the date the message was *sent* and *not* by the date the employee opened the message. For example, an employee who was on vacation or who worked part-time could read all messages delivered during his or her absence on a single day, but the “open” label was attached to the date associated with each email. Three weeks after the close of the 6-month study period, data summarizing opened emails and Web links (by date and participant) were forwarded to study investigators in a spreadsheet. Participants were not aware that these use statistics were an outcome of the study.

The number of emails opened was calculated as the sum of all opened emails over the 6-month (26-week) study period (maximum of 129 messages). To assess sustained participation, the number of days on study was defined as the last date an email was opened minus the first date emails were sent (October 6, 2003). We also calculated the prevalence of opening 4 or 5 daily emails each week as a second measure of sustained, active participation.

### Analysis

The analysis included the tabulation of frequencies, means, and standard deviations and the use of inferential statistics (*t* tests or one-way analysis of variance [ANOVA]) to assess differences in the measures of participation across demographic characteristics. All analyses were conducted in the Statistical Package for the Social Sciences (SPSS). Figures were constructed using DeltaGraph version 5.4.

## Results

### Enrollment

Of the 960 full-time employees, 388 (40%) signed consent forms and enrolled in the study. The baseline health survey was completed by 345 (88%) of the 388 employees who enrolled. Within the first week, 2 of the 345 employees informed study staff that they would be unable to continue in the study due to personal reasons. As shown in Table 1, participating employees were predominantly female (87%) and white (91%), with a mean age of 43.7 years (SD = 8.7). The majority of participants were married, 34% completed college or post-graduate work, 21% earned an annual salary less than $29999, and over half earned less than $39999. With the exception of the gender distribution, characteristics of participating employees did not differ from the total full-time employed population at this worksite—a greater proportion of females enrolled in the study than were employed at the worksite (Table 1).

**Table 1:** Baseline demographic characteristics of participants (n = 345)^*^ and total employee population at the worksite (n = 960)

	**Participants**		**Total Employee Population**
	**N**	**%**	**%**
**Gender**			
Male^†^	44	13	24
Female	299	87	76
**Age (years)**			
20–29	17	5	6
30–39	107	32	31
40–49	128	38	39
50–59	77	22	21
60–66	10	3	3
Mean age (SD)	43.7	8.7	43
Median age	43.3		NA
**Ethnicity**			
White	309	91	90
Black	23	7	8
Other	7	2	2
**Marital Status**			
Married/partner	244	71	66
Divorced/separated/widowed	66	20	NA
Never married	33	9	NA
**Education**			
High school	87	25	NA
Some college	142	41	NA
College graduate	78	23	NA
Postgraduate work	37	11	NA
**Income (US $)**			
< 19999	4	1	2
20000–29999	63	20	22
30000–39999	104	32	26
40000–49999	68	21	20
50000–59999	44	13	13
60000–69999	17	5	6
70000–79999	8	2	2
> 80000	19	6	9

^*^Total numbers do not add to 345 within specific characteristics due to missing data.

^†^ *P* < 0.05 for gender distribution in participant sample compared to total workforce.

NA = not available

**Table 2:** Participation measures at 6 months (n = 345)

	**N**	**%**
Number of Emails Opened^*^		
None	12	3
1–25	35	10
26–51	46	13
52–77	26	8
78–103	40	12
104–117	34	10
118–129	152	44
Mean (SD), Median	88.6 (43.8), 108	
Number of Days on Study^*^^†^		
None	14	4
1–35	4	1
36–71	11	3
72–107	11	3
108–143	13	4
144–161	14	4
162–179	278	81
Mean (SD), Median	159 (44.9), 179	
Number of RealAge Clicks for Additional Information^*^		
None	36	10
1–15	235	68
16–31	42	12
32–51	15	4
52–95	11	3
96–127	2	< 1
128–160	4	1
Mean (SD), Median	13.5 (22.3), 6.0	
**Opened 4 or 5 Emails per Week**		
Never	34	10
1–6 times	55	16
7–12 times	35	10
13–18 times	28	8
19–24 times	59	17
25–26 times	134	39
Mean (SD), Median	16.5 (9.9), 21	

^*^“None” is part of the 1st quintile in measures 1 to 3; in addition, the most populated quintile in measures 1 to 3 has been split at the midpoint to provide more detail about the distribution (5th quintile in measures 1 and 2, 1st quintile in measure 3).

^†^Calculated as the last date an email was opened minus the first date emails were sent.

### Participation

Of the study participants, 3% (n = 12) failed to open any of the 129 email messages, while an additional 5% (n = 16) opened 5 or fewer emails. The mean number of messages opened was 88.6 (SD = 43.8), and the median was 108 messages. More than 118 of the 129 messages were opened by 44% of participants (Table 2); 81% of the participants continued to open emails for 23 weeks or longer (≥ 162 days, Table 2), with more than 50% of them continuing to open emails throughout the 26-week study period. Figure 2 details the number of emails opened weekly by participants. Although there was an initial decline in the number of participants opening 4 or 5 emails per week, the prevalence remained at approximately 60% from 6 weeks into the study until the 25th week.

The use of Web links for additional information was also collected. Approximately 90% of participants sought additional information at least once while enrolled in this study. The mean number of Web links used was 13.5 (SD = 22.3); the median was 6 (Table 2). For context, at least two Web links were embedded in each of the 129 daily messages, offering more than 250 possible Web links over the study course.

**Figure:**
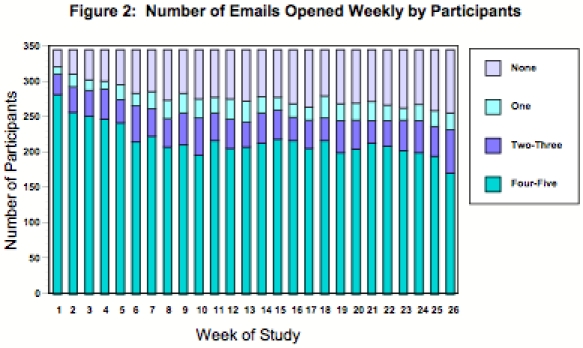
Number of Emails Opened Weekly by Participants

As shown in Table 3, seeking health information via Web links was more frequent among older participants, those with a high school degree, and those with an income of < $40000 per year. Participation (as measured by the overall number of emails opened, the number of days on study, or the number of weeks when 4 or 5 emails were opened) did not differ by gender, age group, ethnicity, marital status, education, or income (Table 3). Additionally, there were no differences in participation or in the number of clicks for additional information as a function of baseline fruit and vegetable intake or physical activity (Tables 4 and 5).

**Table 3:** Study participation at 6 months by baseline demographic characteristics^*^

	**Number of Emails Opened**		**Number of Days on Study**		**Number of Clicks for Additional Information**		**Number of Weeks 4 or 5 Emails Opened**	
	**Mean**	**SD**	**Mean**	**SD**	**Mean**	**SD**	**Mean**	**SD**
**Gender**								
Male	83.9	45.6	158.3	42.4	8.1	19.5	15.5	10.2
Female	89.1	43.6	159.4	45.5	14.3	22.7	16.6	9.9
**Age (years)**								
20–29	96.2	41.5	155.4	47.8	6.0^†^	6.3	18.7	9.2
30–39	87.2	42.5	160.8	40.9	9.8	13.7	16.1	9.7
40–49	88.2	44.5	159.7	46.9	10.8	18.2	16.4	10.1
50–59	92.4	43.6	163.1	39.7	23.5	32.8	17.4	10.1
60–66	64.9	52.4	126.2	75.8	25.0	39.7	11.8	11.5
**Ethnicity**								
White	90.2	42.5	160.9	42.6	13.5	22.1	16.8	9.8
Non-white	74.3	51.4	148.0	58.7	11.9	21.4	13.5	11.2
**Marital Status**								
Married/partner	90.8	42.5	161.1	48.2	12.4	20.1	16.9	9.8
Not married	82.4	46.7	154.9	43.7	16.1	27.1	15.2	10.5
**Education**								
High school	87.5	47.0	156.3	51.9	18.4^†^	27.3	16.5	10.6
Some college	92.2	41.7	159.5	43.2	14.7	24.4	17.4	9.5
College degree	88.7	43.5	159.4	44.4	8.3	13.2	16.6	9.8
Post college	76.6	44.0	166.1	35.5	8.1	9.7	13.1	10.3
**Income (US $)**								
< 39999	90.3	43.9	156.5	48.5	17.0^†^	26.1	17.0	9.9
40000–79999	88.2	43.5	161.8	43.4	9.7	16.1	16.4	9.9
> 80000	72.7	40.4	166.5	27.4	6.2	6.0	11.7	9.1

^*^Total number of messages sent = 129; total number of possible days on study = 179; total number of weeks emails sent = 26

^†^ *P* < 0.05

**Table 4:** Study participation at 6 months by baseline stage-of-change and intention-to-change behaviors^*^

			**Number of Emails Opened**		**Number of Days on Study**		**Number of Clicks for Additional Information**		**Number of Weeks 4 or 5 Emails Opened**	
		**N**	**Mean**	**SD**	**Mean**	**SD**	**Mean**	**SD**	**Mean**	**SD**
**Stage of Change**										
	**Eating 5 daily servings of fruit/vegetables**									
	Yes, for > 6 months	72	93.7	40.9	165.1	38.3	14.2	23.9	17.7	9.5
	Yes, for < 6 months	22	86.2	47.1	145.6	55.9	21.1	22.9	16.1	10.4
	No, start in next 1 month	131	88.7	43.5	160.6	43.2	12.4	21.8	16.6	9.9
	No, start in next 6 months	69	83.6	45.9	153.0	51.3	14.3	23.9	15.3	10.6
	No, do not intend	37	95.0	41.5	167.4	35.4	10.9	20.5	17.9	9.3
	**Getting 30 minutes of daily physical activity**									
	Yes, for > 6 months	71	91.4	41.8	162.5	40.7	16.2	26.7	17.0	9.6
	Yes, for < 6 months	36	91.3	44.6	156.9	44.7	9.2	10.8	17.5	9.9
	No, start in next 1 month	121	87.2	42.9	162.1	41.6	14.9	25.6	16.0	9.9
	No, start in next 6 months	82	87.3	46.2	155.1	51.7	11.4	18.1	16.4	10.4
	No, do not intend	26	83.2	49.2	150.0	55.4	8.9	8.8	15.5	10.9
**Intention to Change**										
	**Eating 5 daily servings of fruit/vegetables**									
	Very unlikely	44	92.2	44.7	162.1	40.6	8.7	8.9	17.5	9.9
	Somewhat unlikely	76	84.9	44.7	158.6	46.9	15.5	26.7	15.6	10.1
	Somewhat likely	147	87.5	44.9	155.2	48.8	11.9	17.7	16.4	10.1
	Very likely	73	92.7	40.3	165.7	38.0	17.4	29.7	17.1	9.7
	**Getting 30 minutes of daily physical activity**									
	Very unlikely	39	89.1	46.5	156.9	51.8	13.2	18.2	16.5	10.6
	Somewhat unlikely	70	88.2	46.8	155.6	49.4	8.7	12.6	16.7	10.2
	Somewhat likely	149	91.8	41.1	162.9	38.6	14.0	20.2	17.2	9.6
	Very likely	79	81.7	45.1	155.6	50.2	17.4	32.6	14.8	10.2

^*^Total number of messages sent = 129; total number of possible days on study = 179; total number of weeks emails sent = 26;*P* > 0.05 in all comparisons

**Table 5:** Study participation by baseline behaviors: servings of fruits and vegetables, and physical activity (n = 345)^*^

			**Number of Emails Opened**		**Number of Days on Study**		**Number of Clicks for Additional Information**		**Number of Weeks 4 or 5 Emails Opened**	
		**N**	**Mean**	**SD**	**Mean**	**SD**	**Mean**	**SD**	**Mean**	**SD**
**Daily Servings of Fruits and Vegetables^†^**													
	< 2	111	84.9	44.3	160.1	43.2	11.9	22.3	15.8	10.0
	3 or 4	102	86.6	45.7	157.3	46.2	11.4	18.7	15.9	10.3
	≥ 5	122	93.7	41.2	160.6	44.9	15.6	22.1	17.6	9.6
**Physical Activity (days/week)**													
	**Vigorous Activity**												
	None	186	89.2	44.6	158.9	45.8	13.2	21.8	16.7	10.1
	1–2	79	87.9	43.7	160.8	43.5	14.3	25.2	16.3	10.1
	3–4	59	88.8	41.6	162.1	39.1	13.1	20.5	16.5	9.6
	5–7	16	83.3	45.1	152.4	57.6	12.7	21.1	15.1	9.9
	**Moderate Activity**												
	None	123	88.7	45.3	155.8	50.9	12.8	23.7	16.8	10.1
	1–2	117	87.2	45.5	157.4	46.4	12.2	19.1	16.2	10.3
	3–4	54	84.0	42.9	163.9	37.9	13.4	24.8	15.0	9.9
	5–7	44	94.9	36.1	169.4	26.3	18.4	23.9	17.6	8.8
	**Walking 10 or more minutes per occasion**												
	None	42	80.6	46.4	155.4	53.6	7.9	8.6	14.9	10.2
	1–2	56	93.9	39.8	163.1	40.1	17.0	30.2	17.7	9.4
	3–4	63	92.4	44.9	159.1	47.2	13.4	23.3	17.4	10.2
	5–7	181	87.3	43.9	159.3	43.8	13.4	20.9	16.2	10.1

Note: Vigorous activity includes heavy lifting, digging, aerobics, fast bicycling, etc. Moderate activity includes bicycling at regular pace, carrying light loads, doubles tennis, etc.

^*^Total number of messages sent = 129; total number of possible days on study = 179; total number of weeks emails sent = 26; *P* > 0.05 in all comparisons

^†^10 outliers eliminated.

More than one third of employees at one large worksite who were enrolled in the study consistently opened health promotion emails throughout the 6-month intervention. Sustained participation was observed for both males and females, across all age groups, education levels, incomes, ethnic groups, and marital status categories. Moreover, participation did not vary by level of baseline health behaviors. The heterogeneity of participants with sustained email open rates supports both the reach and feasibility of an email health promotion program in the workplace.

Of the total workforce at the study worksite, 40% were enrolled in the study. Participants were representative of the total employee sociodemographic profile, with the exception that significantly more women enrolled. This observation is similar to other reports showing that women are more likely than men to participate in face-to-face health promotion programs [17,18]. Diverse age, education, and income levels were represented in the employee population. Wedefined a participant as an employee who completed both an informed consent and a 30-minute health assessment. It is possible that the completion of the lengthy baseline assessment may have discouraged further participation; that is, perhaps a larger proportion of employees would have participated and used worksite health messages if the baseline assessment was not required. It is also possible that we attracted the more motivated employees who did not perceive the assessment as a barrier. However, the varied initial levels of healthy behaviors (i.e., fruit and vegetable intake and physical activity) and readiness-to-change categories (i.e., stage-of-change and intention-to-change) suggest that the email program engaged a heterogeneous employee population with regard to health behaviors and their hypothesized antecedents.

A limitation of traditional health promotion programs is that they attract primarily those who are already motivated to consider health behavior change. Similarly, the frequent users of Internet health websites are more health-oriented than the average population [19]. By using “electronic outreach,” the current email intervention reverses the traditional relationship with health promotion materials. Web-based information was delivered to participants at their desktop, in small, daily email tips, thus eliminating the need for the user to seek out health information, search the Web, or contact a health professional directly. Variation in self-reported health behaviors, stage of change, and intention to adopt health behaviors at the start of the intervention offers evidence that the email program included less-motivated adults. Importantly, we did not detect variation in email use across stage of change or self-reported intention to change. These findings indicate the potential for eHealth promotion programs to reach adults with less than optimal behaviors.

In contrast to the sustained email open rate, fewer participants used the embedded Web links over time, and there was some variability in use by demographic characteristics. It is difficult to interpret these data; we speculate that perhaps people were not interested in general in the additional information (e.g., younger individuals did not feel themselves at risk for health issues), or they were not ready to seek out additional information. It is also possible that those with more education or income had other sources of available information. Alternately, the design and/or content of the Web links may not have appealed to our participants. Future research will need to investigate the factors that influence Web link use.

Longitudinal analyses demonstrated that about 75% of participants “actively” opened messages for 6 months; “actively” included opening a large number of emails or opening fewer emails overall, but continuing to open emails for at least 23 of the 26 weeks. These participants may represent different types of users, a topic worthy of further study. An initial decline in usage occurred from week 2 to 7 of the study. Subsequently, email use remained relatively constant through week 25. Decline in use over time may suggest that participants habituated to the intervention, consistent with the “law of attrition” [18], or that seasonal factors reduced participants’ time for messages. The first third of the study period included both the winter holidays and the business’ peak work season. Further research should evaluate the optimal email interval (e.g., daily vs. weekly) and intervention length in order to optimize employee engagement.

The email program participation rate compares favorably to the 30–40% rates reported for traditional health education and health risk assessment programs [20–22]. However, the unique aspect of this program was the persistent, daily participation across 6 months, longer than a traditional health education program. As expected, this study’s participation rate exceeds the 10–17% rates reported in fitness programs requiring physical participation [20,22].

Successful email health promotion programs may be limited to worksites where regular personal computer access is an expectation. Service industry sites (e.g., financial, educational, marketing) may be particularly appropriate. Settings where Web access is limited or where employees share a computer station (e.g., hospitals, manufacturing plants) may not be easily included in this model. In the future, home email delivery could allow worksite health promotion programs to reach employee families and retirees, in addition to the current workforce.

Based on our findings, we encourage health promotion professionals, employers, and insurers to explore the use of email to deliver health promotion programs. Our results suggest that broad and diverse employee populations can be reached with this technology. The email program we studied sustained use over 26 weeks among varied employee demographic categories. Our ongoing research includes a randomized controlled trial to evaluate both reach and effectiveness (i.e., health behavior change) in a variety of worksite settings. Understanding both reach and effectiveness will allow us to calculate the true public health impact [23] of email and Web health promotion programs. The potential value of eHealth technology to improve active patient participation in health care through information and self-care tools has been well delineated [24,25]. Further research should evaluate message framing, email intervals, duration of intervention, and content of Web supports in order to optimize reach, effectiveness, and, ultimately, public health impact.
